# A Neuronal Hub Binding Sleep Initiation and Body Cooling in Response to a Warm External Stimulus

**DOI:** 10.1016/j.cub.2018.05.054

**Published:** 2018-07-23

**Authors:** Edward C. Harding, Xiao Yu, Andawei Miao, Nathanael Andrews, Ying Ma, Zhiwen Ye, Leda Lignos, Giulia Miracca, Wei Ba, Raquel Yustos, Alexei L. Vyssotski, William Wisden, Nicholas P. Franks

**Affiliations:** 1 Department of Life Sciences, Imperial College London, South Kensington SW7 2AZ, UK; 2 Institute of Neuroinformatics, University of Zürich/ETH Zürich, Winterthurerstrasse 190, 8057 Zürich, Switzerland; 3 Centre for Neurotechnology, Imperial College London, London SW7 2AZ, UK; 4 UK Dementia Research Institute at Imperial College London, London SW7 2AZ, UK

**Keywords:** sleep, thermoregulation, TetTagging, hypothermia, NOS1, torpor, energy balance, sedation, warm stimulus, anesthesia

## Abstract

Mammals, including humans, prepare for sleep by nesting and/or curling up, creating microclimates of skin warmth. To address whether external warmth induces sleep through defined circuitry, we used c-*Fos*-dependent activity tagging, which captures populations of activated cells and allows them to be reactivated to test their physiological role. External warming tagged two principal groups of neurons in the median preoptic (MnPO)/medial preoptic (MPO) hypothalamic area. GABA neurons located mainly in MPO produced non-rapid eye movement (NREM) sleep but no body temperature decrease. Nitrergic-glutamatergic neurons in MnPO-MPO induced both body cooling and NREM sleep. This circuitry explains how skin warming induces sleep and why the maximal rate of core body cooling positively correlates with sleep onset. Thus, the pathways that promote NREM sleep, reduced energy expenditure, and body cooling are inextricably linked, commanded by the same neurons. This implies that one function of NREM sleep is to lower brain temperature and/or conserve energy.

## Highlights

• Nitrergic-glutamatergic neurons in the preoptic area are excited by external warmth
• Reactivation of these neurons induced a drop in body temperature and NREM sleep
• This circuitry binds a warm stimulus, NREM sleep, and body cooling

## Graphical Abstract

**Figure:**
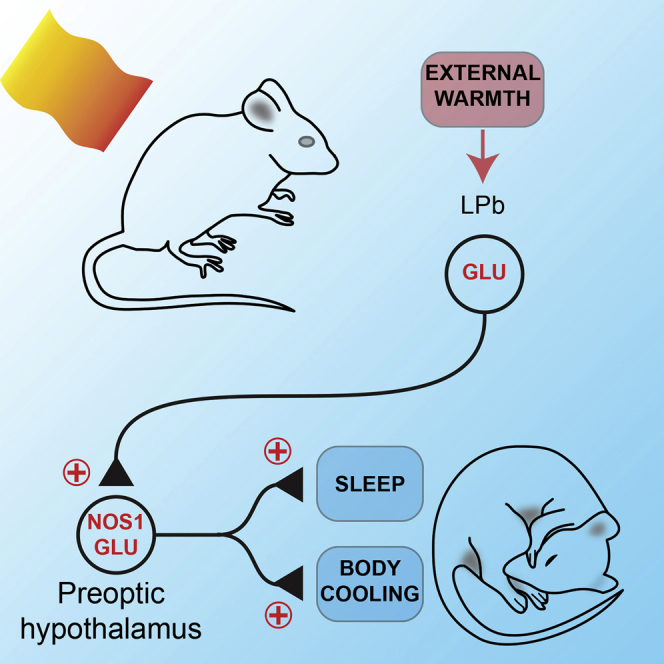

## Teaser

Harding et al. identify nitrergic/glutamatergic neurons in the MnPO-MPO hypothalamus, which after activity tagging by a warm external stimulus induce both sleep and hypothermia when reactivated. This suggests sleep and body cooling are inextricably linked. Reactivating GABAergic neurons, however, induces only sleep.

## Introduction

Specific animal behaviors prior to natural sleep are well-recognized components of sleep initiation. Humans curl up in bed, and rodents and birds go to nests. While central circuits governing the timing of, and switching between, sleep states are being increasingly revealed [1, 2, 3], we are only at the start of analyzing circuitry that might govern how environmental signals trigger sleep onset [2, 4, 5, 6]. Certainly in mice, mild ambient warmth induces sleep [7]. It has been proposed that the reason humans and other mammals prepare for sleep by getting warm with bedding or nesting and also concomitantly lying down is to raise their skin temperature to help trigger sleep [4]. Indeed, hot baths, warm feet, and other localized warming, including, in mice, from brown adipose tissue thermogenesis, all reduce the latency to non-rapid eye movement (NREM) sleep [8, 9, 10, 11, 12, 13]. It is also noteworthy that, in preindustrial tribal peoples, skin warming of the extremities occurs before sleep onset [14]. In other words, for mammals, raising skin temperature prior to sleep could be, under some circumstances, a sleep-permissive condition and part of a natural mechanism to induce sleep [4, 15]. Getting warm prior to sleep, however, could also simply be a matter of comfort rather than a specific mechanism. Furthermore, no circuitry has yet been described that could directly allow external warmth to induce NREM sleep.

Both sleep and temperature regulation converge in the preoptic (PO) hypothalamus [16, 17, 18, 19]. This region has a heterogeneous and complex mix of neurons that also command homeostatic responses for electrolyte balance, energy expenditure, sexual responses, and the cardiovascular system [18, 20]. It has been generally assumed that the neurons in the preoptic regulating sleep and body temperature are separate. Neurons in the preoptic, when directly heated, can induce NREM sleep [18]. But it is unlikely that this is the physiological mechanism that would induce sleep following mild external warming of the body—in fact, there is no clear relationship between external skin temperature and brain temperature [17, 21]. Thus, we need to consider other possibilities for how mild warming might promote sleep.

As well as inducing NREM sleep, sustained external warmth to the skin induces a homeostatic response to cool the body [16, 22]. An excitatory glutamatergic pathway runs from sensory neurons in the skin, through cells in the spinal cord, glutamatergic relay neurons in the lateral parabrachial nucleus, and then up to the median (MnPO) and medial (MPO) preoptic areas of the hypothalamus [16, 22, 23, 24]. The precise circuitry within the preoptic area that responds to increased external warmth by receiving the excitatory input from the lateral parabrachial nucleus is still being elucidated [19]. Preoptic neurons command descending pathways to switch off heat production in brown adipocytes, promote vasodilation, and induce drinking [16, 21, 22, 25, 26, 27, 28].

In this paper, we use the genetic technique of “activity tagging” to identify MPO-MnPO hypothalamic neurons that respond to a warm stimulus but which, when reactivated, simultaneously induce both sleep and a reduction in body temperature, thus showing that sleep induction and body cooling are inextricably linked.

## Results

### Mice Prefer a Warm Environment for Nesting prior to Sleep

**Figure 1:**
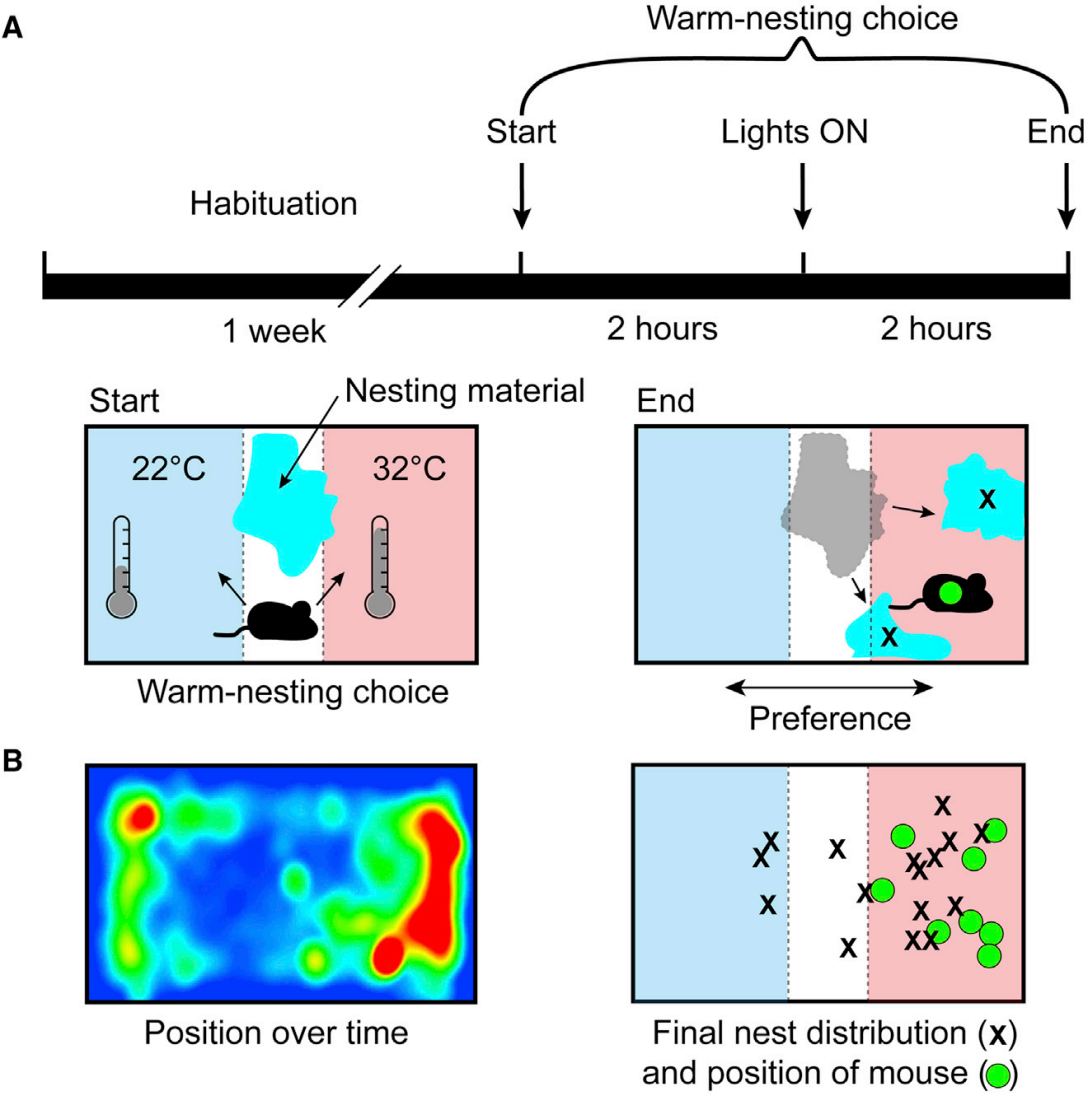
Temperature Preference Test: Mice Prefer to Locate to a 32°C Zone (A) Protocol for testing temperature preference. A mouse was allowed to habituate in a home cage with nesting material (cyan) at ambient temperature (22°C ± 1°C) for one week. At the end of the habituation period, and two hours before “lights on,” temperature-controlled plates were turned on such that one end of the cage was set at 32°C ± 1°C while the other end remained at the ambient temperature (left schematic). Two hours after lights on, the final position of the mouse and the distribution of the nesting material were recorded (right). (B) (Left) A record of the average positions of the mice during the two hours after lights on and (right) the final position of each mouse (n = 8; green circles) and the positions of the two largest fragments of nesting material (black crosses).

Laboratory mice are usually housed at temperatures (20°C–24°C) significantly below the temperature at which their metabolic expenditure is at a minimum, the so-called “thermo-neutral zone”. In mice, the thermo-neutral zone can range from 26°C to 34°C [29]. Given the choice, mice will move to such warmer environments [30]. We first confirmed that our warm stimulus of 32°C for genetic activity tagging provided a preferred environment for mice compared with keeping them at an ambient temperature of 22°C. To do this, we allowed mice to habituate during one week in a cage at 22°C with bedding material, with which they built nests (Figure 1A). At the end of this habituation period, and 2 hr before “lights on,” we used thermostatically controlled plates on the base of the cage to warm one end of the cage to 32°C while the other end was set at the ambient temperature of 22°C and collected the bedding material and placed it in the center of the cage (Figure 1A). We then measured the position of the mice over the 2 hr after lights on and the final position of the mice and the bedding material (Figure 1B). We found that mice (n = 8) spent 73% ± 4% of their time in the warm end of the cage (Figure 1B, left), and this preference was reflected in both the final position of the mice and the distribution of bedding material (Figure 1B, right).

### A Warm Stimulus Induces c-FOS Expression in the Preoptic Hypothalamus, which Can Be Used to Activity Tag Neurons

**Figure 2:**
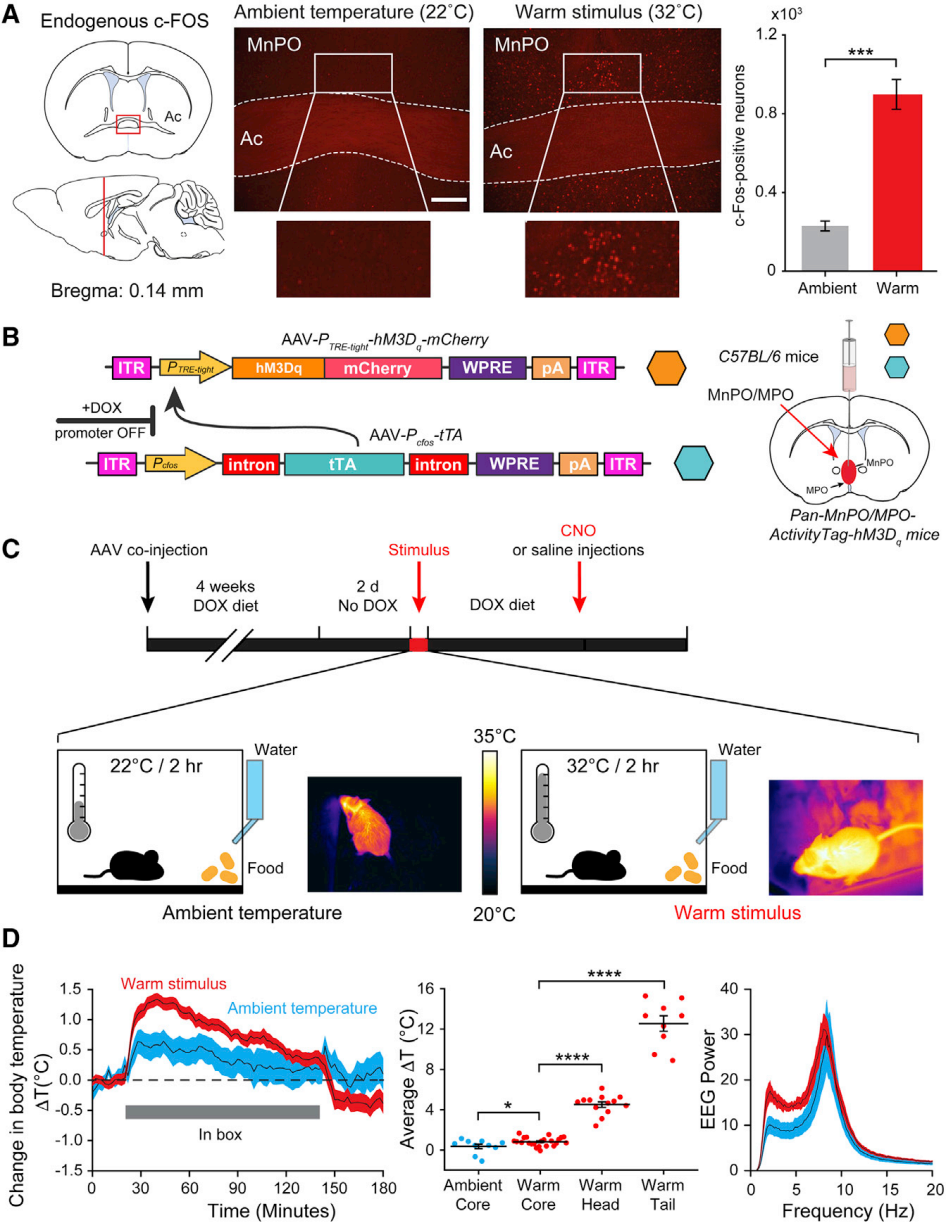
Neurons in the Hypothalamus Can Be Activity Tagged Using a c-*fos* Promotor (A) Compared with the expression after exposure of the mice to an ambient temperature of 22°C ± 1°C (left image), a warm stimulus of 32°C ± 1°C induces robust c-Fos expression throughout MnPO (right image) and MPO (Figures S1A and S1B). The graph on the right shows that a warm stimulus induces c-Fos expression in a significantly higher number of neurons compared to ambient temperatures (p = 1.57 × 10^−4^; df = 6; n = 4 mice per group). Ac, anterior commissure. The scale bar represents 200 μm. ^∗∗∗^p < 0.001. (B) The AAV transgenes used to activity tag warm-sensitive neurons. The hM3D_q_-mCherry fusion protein is expressed, driven by the *P*_*TRE-tight*_ promoter, but only after neuronal activity has driven expression (by the *c-fos* promotor) of the tetracycline transactivator protein (tTA) and only in the absence of doxycycline (DOX). AAVs containing the transgenes were injected on the midline into the median and medial preoptic (MnPO and MPO) hypothalamus to generate *Pan-MnPO/MPO-ActivityTag-hM3D*_*q*_ mice. ITR, inverted terminal repeats; pA, polyadenylation signal; WPRE, woodchuck-posttranscriptional-regulatory element. (C) Protocol and timeline for the activity-tagging experiments. Two days after doxycycline (200 mg/kg) was removed from the diet, the *Pan-MnPO/MPO-ActivityTag-hM*_*3*_*D*_*q*_ mice were placed in a box at a warm temperature of 32°C ± 1°C or at the ambient temperature of 22°C ± 1°C for 2 hr. (D) During this time (left and center), their average core body temperature increased by 0.8°C ± 0.09°C (n = 23) and 0.4°C ± 0.2°C (n = 10). During the warm stimulus, the temperature of the skin on the head increased by 4.5°C ± 0.3°C (n = 13) to 35.2°C ± 0.2°C, and the skin temperature measured on the tail increased by 12.5°C ± 0.8°C (n = 9) to 35.6°C ± 0.4°C. Under both warm and ambient temperatures, the Fourier transform power spectra (right) showed a waking EEG, with maxima at theta frequencies. The error bars in (A) and (D) represent the SEM, as do the error envelopes in (D). ^∗^p < 0.05; ^∗∗∗∗^p < 0.0001. See also Figure S1.

Mice showed a clear preference for exhibiting behaviors that involve preparing to sleep at a temperature close to their thermo-neutral zone [29]. Neurons in the MnPO and MPO areas also respond to external environmental warming by expressing the *c-fos* gene [25, 31] (Figures 2A, S1A, and S1B). This is a selective process—the neocortex, for example, does not have increased numbers of cFOS-positive neurons following external warming (Figures S1C and S1D). We therefore used *c-fos*-based activity tagging [32], also known as TetTagging [33], to drive a c-*fos*-promoter-dependent pulse of *hM3D*_*q*_*-mCherry* receptor expression in the MnPO-MPO area during exposure of the mice to an increase in ambient temperature. This method allowed us to test the hypothesis that a warm stimulus elicits NREM sleep via the MnPO-MPO hypothalamus.

We first validated in cultured HEK293 cells that the activity-tagging transgene system (*P*_*cfos*_*-tTA* and *P*_*TRE-tight*_-*hM3D*_*q*_*-mCherry*) was tightly regulated by doxycycline (Figure S2A). In this system, the *c-fos* promoter has high basal activity because of serum components in the media and cell division, and thus tTA from the *P*_*cfos*_*-tTA* transgene is present; nevertheless, doxycycline efficiently repressed hM3D_q_-mCherry expression (Figure S2A). Removing doxycycline from the media, and so allowing the tTA protein to drive transcription of the hM3D_q_-responsive transgene *P*_*TRE-tight*_-*hM3D*_*q*_*-mCherry*, produced a large increase in hM3D_q_-mCherry expression (Figure S2A).

Next, we injected the activity-tagging system (adeno-associated virus [AAV]-*P*_*cfos*_*-tTA* and AAV-*P*_*TRE-tight*_-*hM3D*_*q*_*-mCherry*) into the MnPO and MPO hypothalamic area of *C57BL/6* mice to generate *Pan-MnPO/MPO-ActivityTag-hM3D*_*q*_ mice (“*Pan*” designates that all c-FOS-expressing cell types are captured by the *c-fos-*dependent tagging) (Figure 2B). The *hM3D*_*q*_*-mCherry* transgene, under tTA control, was suppressed by doxycycline. To allow activation of the transgene, the doxycycline was removed from the diet for two days (Figure 2C), and the mice were placed in a box (at zeitgeber time 21:00) for 2 hr at 32°C (Figure 2C) or at the ambient temperature of 22°C. During these 2 hr at 32°C or 22°C, the average core body temperature of the mice increased by only 0.8°C ± 0.09°C (n = 23) and 0.4°C ± 0.2°C (n = 10), respectively (Figure 2D). However, the skin temperature measured on the surface of the head increased by 4.5°C ± 0.3°C (n = 13) to 35.2°C ± 0.2°C, and the skin temperature measured on the tail increased by 12.5°C ± 0.8°C (n = 9) to 35.6°C ± 0.4°C (Figure 2D). Under both warm and ambient temperatures, the Fourier transform power spectra (Figure 2D, right) showed a waking electroencephalogram (EEG), with maxima at theta frequencies.

### Reactivation of Neurons of Warm-Stimulus-Tagged Neurons in the MnPO-MPO Region of the Hypothalamus Induces Both a Drop in Body Temperature and Sleep

**Figure 3:**
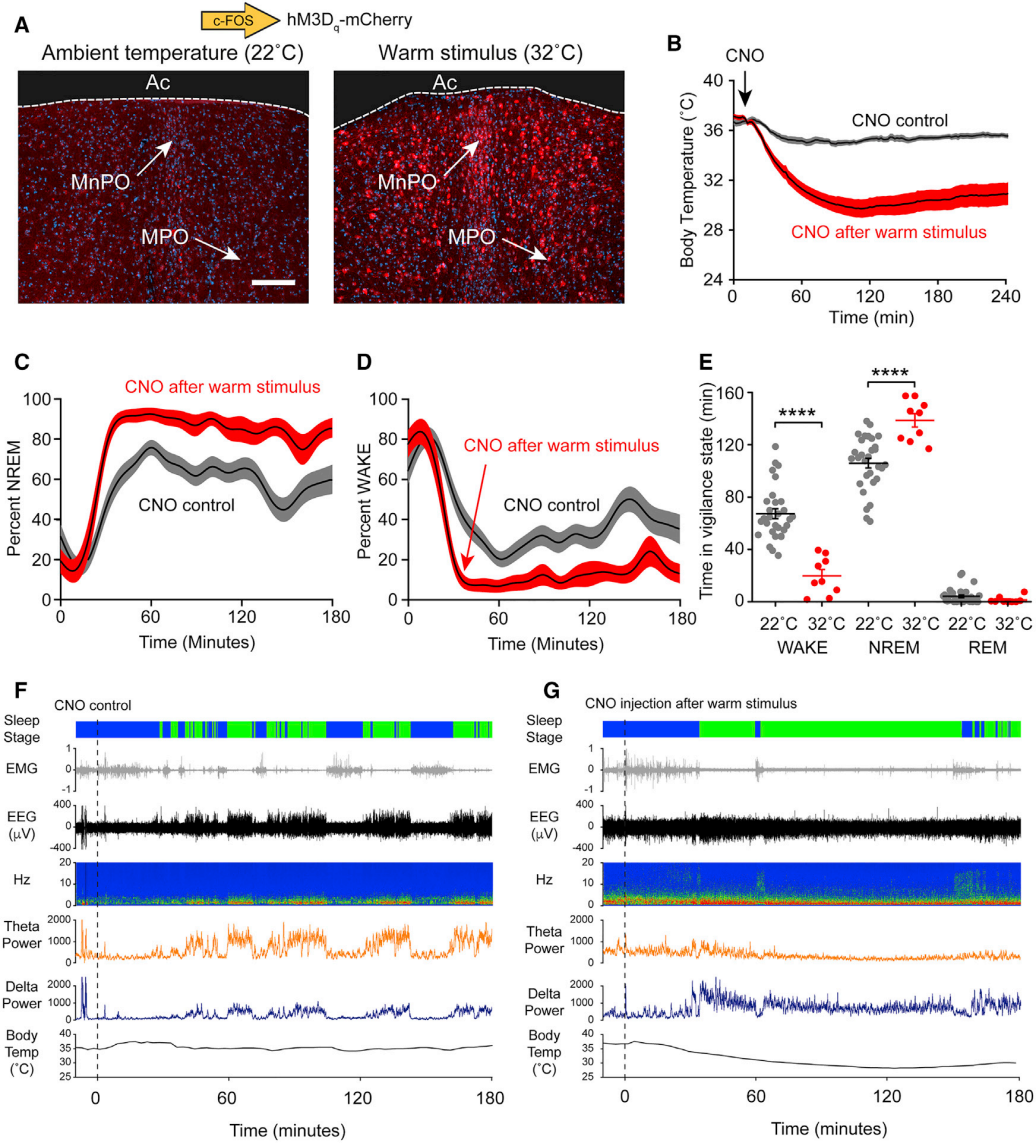
Reactivation of Neurons of Warm-Stimulus-Tagged Neurons in the MnPO-MPO Region of the Hypothalamus Induces Both a Drop in Body Temperature and Sleep (A) After exposure of mice to a warm stimulus (32°C ± 1°C for 2 hr) there was strong *hM3D*_*q*_*-mCherry* expression (red) throughout MnPO and MPO compared to baseline levels of expression seen at ambient temperatures (22°C ± 1°C). DAPI staining is in blue. The scale bar represents 200 μm. (B) When later injected with CNO (5 mg/kg), the warm-stimulated animals (red) exhibited marked reduction in body temperature (n = 14), in contrast to animals (gray) injected with CNO after being exposed to ambient temperatures (n = 13). (C) CNO rapidly induced a state of NREM in animals (n = 9) previously exposed to a warm stimulus (red) compared to animals injected with CNO following exposure to ambient temperatures (gray; n = 30). (D) Animals (n = 9) exposed to CNO following an earlier warm stimulus had minimal waking (red) compared to animals injected with CNO following exposure to ambient temperatures (gray; n = 30). (E) The times in each vigilance state, NREM, WAKE, and REM, are shown after CNO injection after exposure to ambient temperatures (gray) and after CNO injection following exposure to a warm stimulus (red). CNO caused a significant decrease in WAKE times (two-way ANOVA; p = 5.4 × 10^−12^; *df* = 37) and a significant increase in NREM times (two-way ANOVA; p = 5.2 × 10^−7^; *df* = 37) but no change in REM times (two-way ANOVA; p = 0.66; *df* = 37). ^∗∗∗∗^p < 0.0001. (F) Representative example of EEG (electroencephalogram), EMG (electromyogram), and sleep-stage scoring after a CNO injection following ambient temperature exposure in *Pan-MnPO/MPO-ActivityTag-hM3D*_*q*_ mice. During the 3 hr following the CNO injection, the body temperature did not change, and this individual mouse was scored as being in the waking state 43.6% of the time (dark blue), in NREM sleep 56.4% of the time (green), and REM sleep 0.0% of the time. (G) As in (C) but following exposure to a warm stimulus. During the 3 hr following the CNO injection, the body temperature dropped to a minimum of 28°C, and this individual mouse was scored as being in the waking state 25.2% of the time, in NREM sleep 74.7% of the time, and REM sleep 0.2% of the time. The error envelopes in (B)–(D) represent the SEM, as do the error bars in (E). See also Figures S2, S3, S4, and S5.

We examined whether neurons in MnPO and MPO became genetically tagged with *c-fos*-dependent *hM3D*_*q*_*-mCherry* transgene expression following exposure of the mice to warm or ambient stimuli. Compared with *hM3D*_*q*_*-mCherry* expression after ambient temperatures (Figure 3A, left image), the warm stimulus induced robust *hM3D*_*q*_*-mCherry* transgene expression throughout the MnPO and MPO area (Figures 3A, right image, S2C–S2E, and S3). A detailed histological analysis across the preoptic area of multiple animals (n = 11) confirmed that the *c-fos*-dependent *hM3D*_*q*_*-mCherry* transgene induction was restricted to the MnPO-MPO area (Figure S3B), with no expression in the neighboring LPO area (Figure S3C). After the warm and ambient stimuli, the two groups of *Pan-MnPO/MPO-ActivityTag-hM3D*_*q*_ mice were placed back on doxycycline (200 mg/kg) to repress the *P*_*TRE-tight*_-*hM3D*_*q*_*-mCherry* transgene (Figure 2C). Previously, we found that the pulse of synthesized hM3D_q_-mCherry receptor induced by activity tagging persists on tagged neurons for at least a week [32]. This longevity of the receptor enables the mice to recover and be injected with Clozapine N-oxide (CNO) three days after the initial stimulus.

We looked at the physiological consequences of exposure of the mice to warm stimuli by reactivating the tagged MnPO and MPO neurons with systemic CNO and simultaneously recording core body temperature and EEG (Figures 3 and S4). When the mice that had received a 32°C stimulus were injected with CNO (5 mg/kg), they exhibited hypothermia over several hours (the minimum core body temperature reached was approximately 28.7°C ± 0.7°C; n = 14; Figures 3B and S4A). In contrast, when CNO was injected into *Pan-MnPO/MPO-ActivityTag-hM3D*_*q*_ mice that had only been exposed to ambient temperatures, no sustained hypothermia was elicited, although there was a small drop in core body temperature consistent with background activity of the *c-fos* promoter (Figures 3B and S4A).

Concurrently with the start of the temperature decrease, CNO rapidly (after ∼20 min) induced a state of almost constant NREM-like sleep in the warm-exposed *Pan-MnPO/MPO-ActivityTag-hM3D*_*q*_ mice, with minimal waking (Figures 3C–3E and S4B). Some sleep also occurred in the CNO-injected mice that were tagged at ambient (22°C) temperature (Figure 3C), consistent with some background *c-fos* promoter activity, but this amount of sleep was significantly below that evoked following tagging after warming (Figures 3C and 3E). In the mice that had been tagged at 32°C, the CNO-evoked sleep onset occurred before the maximum temperature decrease. This NREM sleep-like state persisted for several hours. As the hypothermia progressed from 37°C to its average nadir of ∼29°C, there was an overall ∼40% reduction in EEG amplitude and a similar percentage reduction in peak delta (1–4 Hz) frequency (Figures 3G, S5A, and S5B). These shifts in the power spectra are because temperature decreases the rate constants of the various ionic processes that produce the EEG [34]. Even in this lower power EEG state, however, the mice could still clearly be scored as being in a NREM vigilance state (high ratio of delta to theta power in the EEG paired with little activity in the EMG; see Figures 3F and 3G), as reported previously [34]. As a control for the specificity of CNO’s actions [35], we confirmed that CNO (5 mg/kg) systemically injected into non-AAV injected naive mice did not induce NREM sleep above the background of sleep occurring following saline injection (Figure S4C). Thus, activity tagging shows that a warm stimulus activates MnPO-MPO neurons, which then induce both hypothermia and NREM sleep.

### A Warm Stimulus Activates Nitrergic-Glutamatergic and GABAergic Neurons in the MnPO-MPO Area

**Figure 4:**
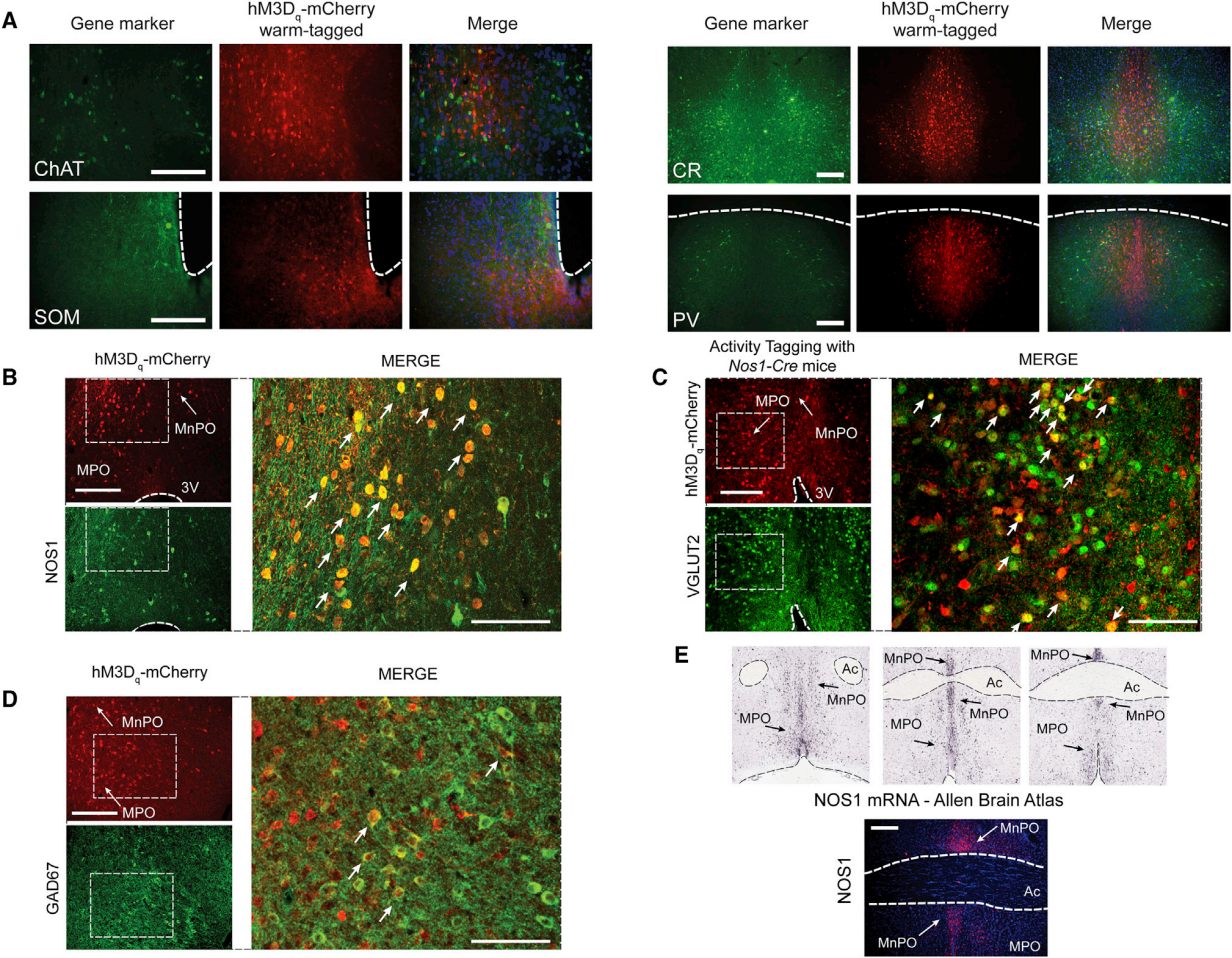
Survey of Neurochemical Markers for Neurons Activity Tagged by External Warmth in the MnPO-MPO Area Double-label immunohistochemistry of warm-labeled (activity-tagged) neurons (red) in the preoptic hypothalamus of *Pan-MnPO/MPO-ActivityTag-hM3D*_*q*_ mice with candidate gene markers (green). (A) Choline acetyltransferase (ChAT), somatostatin (SOM), calretinin (CR), and parvalbumin (PV) all stained negative. The scale bars represent 200 μm. (B) NOS1 antibody (green) stained approximately 25% of hM3D_q_-mCherry-positive neurons (red), indicating nitrergic neurons. Examples of double-labeled cells are indicated with arrows. (C) Many warm-activated *Nos1*-Cre neurons (red) in the MnPO and MPO areas co-stain with VGlut2 antisera (green). Examples of double-labeled cells are indicated with arrows. (D) Glutamate decarboxylase 67 (GAD67) antibody (green) stained approximately 30% of hM3D_q_-mCherry-positive neurons (red), indicating GABAergic neurons. Examples of double-labeled cells are indicated with arrows. (E) The mouse MnPO and MPO hypothalamic area has a population of neurons with strong *Nos1* expression, as visualized by *in situ* hybridization with a *Nos1*-selective probe (top three images), taken from the Allen Brain Atlas [36] and immunohistochemistry with a NOS1 antibody (lower image). In the images on the left of (B)–(D), the scale bars represent 200 μm, and for the larger images on the right of each panel the scale bars represent 100 μm. The scale bar in (E) represents 200 μm.

We next investigated the neurochemical identity of the MnPO-MPO neurons that were activity tagged with the hM3D_q_-mCherry protein. We double stained the MnPO-MPO area in activity-tagged mice with mCherry antisera and a panel of antibodies for neurochemical markers: choline acetyl transferase (ChAT), somatostatin (SOM), calretinin (CR), parvalbumin (PV), neuronal nitric oxide synthase (NOS1), vesicular glutamate transporter 2 (VGLUT2), and glutamic acid decarboxylase 67 (GAD67) (Figure 4). The tagged hM3D_q_-mCherry-expressing neurons were negative for ChAT, SOM, CR, and PV (Figure 4A) but did stain for NOS1 (Figure 4B), VGLUT2 (Figure 4C), and GAD67 (Figure 4D). There were only a few warm-tagged NOS1 cells in MnPO that co-stained with GAD67 (only 56 double positives in 2,018 counted neurons). The warm-tagged GABAergic cells were located mainly in MPO (Figure 4D). However, the majority (56%) of NOS1-tagged neurons co-stained for VGLUT2 (Figure 4C), suggesting that these NOS1 neurons in MnPO are glutamatergic. The Allen Brain Atlas of transcript expression (Figure 4E, top three images), and our own immunocytochemical stainings (Figure 4E, lower image), revealed that the MnPO-MPO area is particularly enriched with NOS1-positive neurons [36].

### Reactivating Warm-Stimulus-Tagged Nitrergic-Glutamatergic Neurons in the MnPO-MPO Hypothalamus Induces Both Sleep and Hypothermia, whereas Tagged GABAergic Neurons in MPO Produce Only NREM Sleep

**Figure 5:**
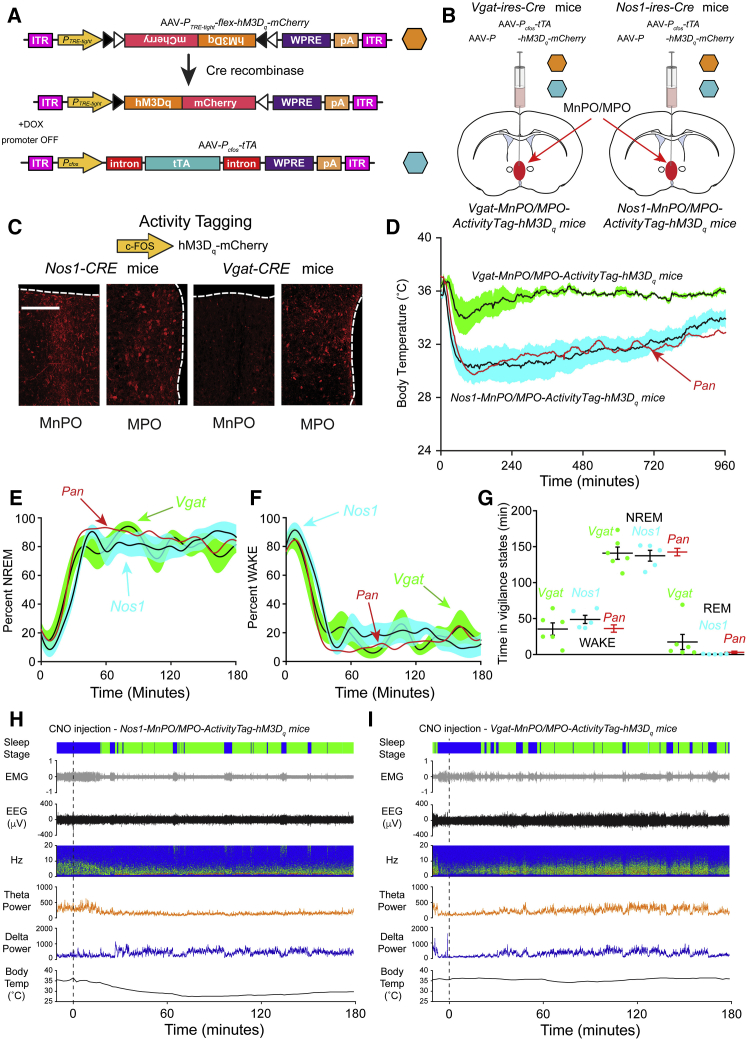
Cell-Type-Selective Activity Tagging Reactivating warm-stimulus-tagged nitrergic neurons in the MnPO-MPO hypothalamus induces both sleep and hypothermia, whereas reactivating warm-stimulus-tagged GABAergic neurons in the MnPO-MPO hypothalamus induces only sleep. (A) The AAV transgenes used to selectively activity tag genetically defined (Cre-positive) and warm-sensitive neurons. The *P*_*TRE-tight*_ promoter (TRE) responds to the *c-Fos*-promoter-controlled tetracycline transactivator protein (tTA) to drive *hM3D*_*q*_*-mCherry* transgene expression restricted to Cre-positive cells and only in the absence of doxycycline. (B) Schematic illustrating midline injection of the two AAV constructs into MnPO-MPO in *Vgat-ires-Cre* mice and *Nos1-ires-Cre* mice to produce *Nos1-MnPO/MPO-ActivityTag-hM3D*_*q*_ mice and *Vgat-MnPO/MPO-ActivityTag-hM3D*_*q*_ mice, respectively. (C) Differential distribution of warm-tagged *Nos1-Cre* neurons and warm-tagged *Vgat-Cre* neurons in MnPO and MPO. The tagged *Nos1* neurons are in both MnPO and MPO areas, whereas the tagged *Vgat* neurons are mainly in MPO area as determined by mCherry immunohistochemistry to detect the hM3D_q_-mCherry receptor. The scale bar represents 150 μm. (D) When later injected with CNO, the warm-stimulated *Nos1-MnPO/MPO-ActivityTag-hM3D*_*q*_ mice exhibited marked hypothermia, which lasted for several hours (n = 5) and which was indistinguishable from that seen in *Pan-MnPO/MPO-ActivityTag-hM3D*_*q*_ mice (shown in red—data from Figure 3B). In contrast, *Vgat-MnPO/MPO-ActivityTag-hM3D*_*q*_ mice injected with CNO showed more modest and transient hypothermia (n = 6). (E) With animals previously exposed to a warm stimulus, CNO rapidly induced a state of NREM in both *Nos1-MnPO/MPO-ActivityTag-hM3D*_*q*_ (blue) and *Vgat-MnPO/MPO-ActivityTag-hM3D*_*q*_ (green) mice. In both cases, cell-type-selective tagging elicited NREM sleep that was not significantly different to pan-tagging in the *Pan-MnPO/MPO-ActivityTag-hM3D*_*q*_ mice (shown in red—data from Figure 3C). (F) With animals previously exposed to a warm stimulus, CNO rapidly induced a state of minimal waking in both *Nos1-MnPO/MPO-ActivityTag-hM3D*_*q*_ (blue) and *Vgat-MnPO/MPO-ActivityTag-hM3D*_*q*_ (green) mice. In both cases, cell-type-selective tagging elicited a waking state sleep that was not significantly different to pan-tagging in the *Pan-MnPO/MPO-ActivityTag-hM3D*_*q*_ mice (shown in red—data from Figure 3D). (G) The times in each vigilance state, NREM, WAKE, and REM, are shown after CNO injection into *Nos1-MnPO/MPO-ActivityTag-hM3D*_*q*_ mice (blue; n = 5) and into *Vgat-MnPO/MPO-ActivityTag-hM3D*_*q*_ mice (green; n = 6). The times for the *Pan-MnPO/MPO-ActivityTag-hM3D*_*q*_ mice are shown for comparison (shown in red—data from Figure 3E). (H) Representative example of EEG, EMG, and sleep-stage scoring after a CNO injection (5 mg/kg) in *Nos1-MnPO/MPO-ActivityTag-hM3D*_*q*_ mice following a warm stimulus. During the 3 hr following the CNO injection, the body temperature dropped to a minimum of 27.5°C, and this individual mouse was scored as being in the waking state 20.0% of the time, in NREM sleep 79.9% of the time, and REM sleep 0.1% of the time. (I) Representative example of EEG, EMG, and sleep-stage scoring after a CNO injection (5 mg/kg) in *Vgat-MnPO/MPO-ActivityTag-hM3D*_*q*_ mice following a warm stimulus. During the 3 hr following the CNO injection, the body temperature dropped to a minimum of 34.2°C, and this individual mouse was scored as being in the waking state 29.1% of the time (dark blue), in NREM sleep 70.6% of the time (green), and REM sleep 0.3% of the time. Data are expressed as mean ± SEM.

We next aimed to examine and differentiate any specific roles of the *Nos1*-expressing and GABAergic neuronal populations in responding to external warmth and producing the hypothermia and NREM sleep. The pan activity-tagging method does not differentiate between subtypes of neurons. To address this, we restricted the activity tagging to genetically specified cell types (see STAR Methods and Figure 5), so that the expression of the *P*_*TRE-tight*_-*hM3D*_*q*_*-mCherry* transgene depended on both Cre recombinase *and* neural activity.

The two activity-tagging AAVs, AAV-P_cFos_-tTA and AAV-P_TRE-tight_-flex-hM3D_q_-mCherry, were co-injected into the MnPO-MPO of *Vgat-ires-Cre* or *Nos1-ires-Cre* mice (Figures 5A and 5B) to generate *Vgat-MnPO-MPO-ActivityTag-hM3D*_*q*_ and *Nos1-MnPO-MPO-ActivityTag-hM3D*_*q*_ mice, respectively. This allowed for the cell-type-selective tagging of GABAergic or nitrergic cells. The activity-tagging protocol with external warming was then repeated exactly as before (Figures 2B and 2C). External warming induced hM3D_q_-mCherry expression in Nos1-Cre cells in MnPO and MPO (Figure 5C), whereas in Vgat-Cre neurons, hM3D_q_-mCherry induction was mainly restricted to the MPO area (Figure 5C).

After placing the *Nos1-MnPO/MPO-ActivityTag-hM3D*_*q*_ and *Vgat-MnPO/MPO-ActivityTag-hM3D*_*q*_ animals back on doxycycline to ensure an isolated pulse of hM3D_q_-mCherry receptor, CNO was administered a few days later and the core body temperature and vigilance states recorded. In *Nos1-MnPO/MPO-ActivityTag-hM3D*_*q*_ mice, CNO caused both a strong and sustained decrease in core body temperature (to the same extent as that produced with *Pan-MnPO/MPO-ActivityTag-hM3D*_*q*_ mice) (Figure 5D) and sustained NREM sleep (Figures 5E–5H). By contrast to the large temperature response of the reactivated warm-tagged *Nos1* neurons, CNO given to *Vgat-MnPO/MPO-ActivityTag hM3D*_*q*_ mice produced only a small and transient hypothermia (Figure 5D) but a sustained NREM sleep equivalent to that obtained with the *Nos1-MnPO/MPO-ActivityTag-hM3D*_*q*_ mice (Figures 5E–5G and 5I).

## Discussion

**Figure 6:**
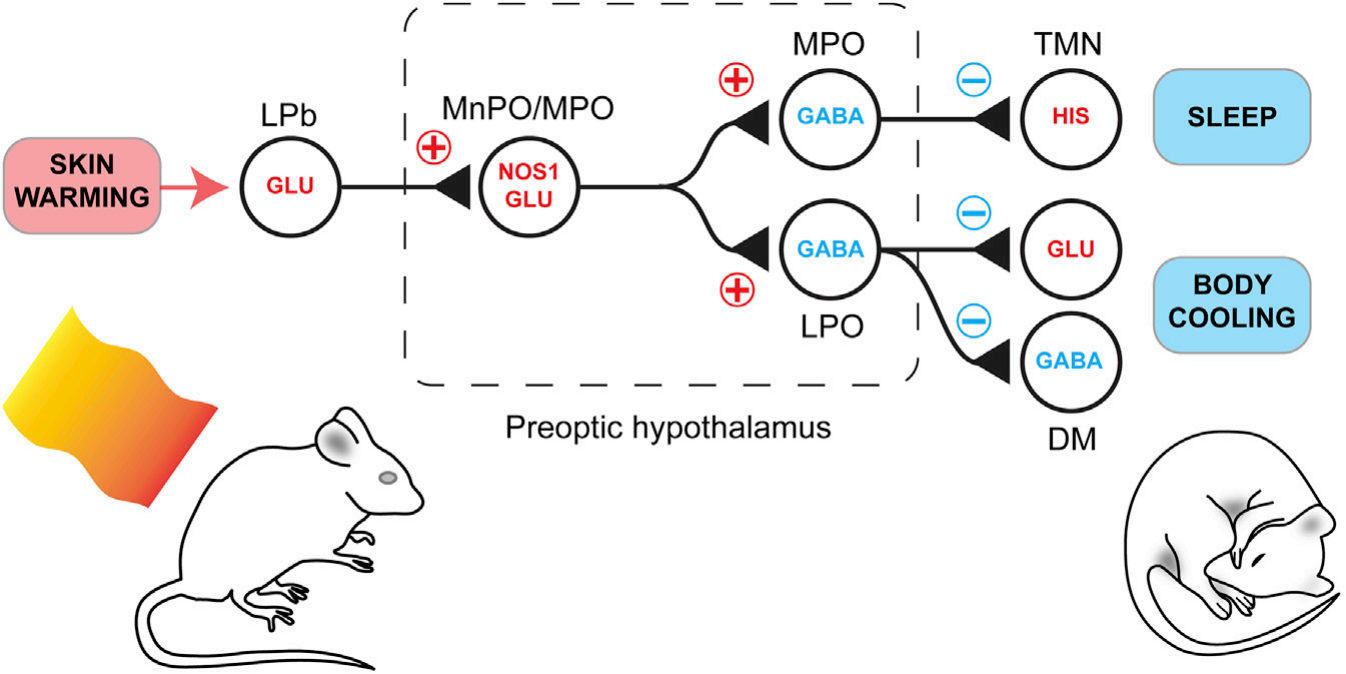
Conceptual Circuit Model for How External Warming Induces and Links Sleep and Body Cooling Skin warming activates a pathway that runs through the spinal cord and is relayed by glutamatergic neurons in the lateral parabrachial nucleus [16, 22, 23]. This activates the *Nos1*-positive glutamatergic neurons in MnPO-MPO whose output drives GABAergic neurons in both MPO and GABAergic neurons in the lateral preoptic (LPO) areas [25]. Others have demonstrated that activating glutamatergic cells in MnPO-MPO induces hypothermia [26, 28] and that GABAergic neurons in the MnPO-MPO area do not regulate body temperature [25, 28]. We suggest the MPO GABA neurons inhibit monoaminergic arousal pathways, such as the histaminergic tuberomammillary nucleus, resulting in sleep; by contrast, the LPO GABA neurons inhibit both heat-promoting glutamatergic and GABAergic neurons in the dorsomedial hypothalamus (DM), resulting in body cooling [25].

Except in the extreme case of fever [37], current descriptions of sleep-promoting circuitry have generally neglected the role of body temperature. In this study, we examined the circuitry underlying how a comfortable stimulus for a mouse, an increase to an ambient temperature in the thermo-neutral zone [29], could lead directly to NREM sleep. We find that NREM sleep induction and body cooling are inextricably linked following a warm stimulus. The activity-tagging method allowed us to selectively isolate and reactivate nitrergic/glutamatergic neurons in the MnPO-MPO hypothalamic area that respond to external warmth and show that these cells trigger both NREM sleep and body cooling. Using the same method, we also identified GABAergic cells in MPO that respond to warming by inducing NREM sleep but without causing body cooling, suggesting that these neurons are downstream of the nitrergic/glutamatergic neurons (Figure 6). The circuit identified here could form part of a mechanism allowing mammals to prepare for sleep.

Although we have discussed our results in the context of external warmth, we cannot separate the effects due to the small increase in body temperature from those caused by the larger increase in skin temperature. Nonetheless, we suggest that external warming elicits sleep and body cooling primarily via skin warming. When the mice are exposed to the ambient warm stimulus of 32°C, the temperatures on the surfaces of the head and tail increase by approximately 4.5°C and 12.5°C, respectively. These increases are probably due to both passive heating as well as heating due to vasodilation, because the skin temperature exceeds that of the external warm stimulus. In contrast, the rise in core body temperature, although it cannot be ignored, is much smaller (∼0.4°C above controls). Even so, more work is required, for example by blocking pathways from peripheral thermosensors, to definitively show that skin warming is driving the circuitry that we have identified.

A methodological point concerns the drop in body temperature that is produced by CNO reactivating the MnPO-MPO nitrergic/glutamatergic neurons. This decrease was considerably larger than that during natural NREM sleep. This over-activation of hypothalamic circuitry with pharmaco- or optogenetics to produce hypothermia (or hyperthermia) was similarly observed in other studies [17, 25, 28]. While pharmaco- and optogenetics are powerful tools for revealing novel circuitry involved in a physiological process, the circuits activated by these methods might not be driven to this extent under normal physiological circumstances. Nonetheless, the hypothermia that we observe of about 10°C is similar to the shallow torpor seen in mice following fasting at room temperature [38]. It is possible that the hypothermia observed under these natural conditions is driven by the same circuitry that we have described here.

Although our data are for mice and might not be reliably extended to humans, our findings on circuitry linking NREM sleep induction and body temperature decreases could explain the classical data, known for many years, that NREM sleep is associated with lower core body and brain temperatures [12, 39]. This is strikingly illustrated in forced desynchrony experiments in humans, where the circadian variation in body temperature can be uncoupled from NREM sleep episodes so that sleep can occur at any point on the circadian temperature cycle [40]. In this scenario, body temperature always falls during the entry into NREM sleep, even if the circadian body temperature is on the rising part of its curve (see Figure 5E in [40]). Similarly, by far the biggest determinant of brain temperature is vigilance state rather than circadian phase [39].

For humans, temperature is an important determinant of sleep duration and timing [14]. During wakefulness, the core body temperature is higher and the skin temperature is lower; on the other hand, during NREM sleep, the opposite pertains [41]. Before sleep onset, distal skin temperature rises in humans [14, 41], and a high proximal-to-distal temperature gradient of the skin, such as feet warming, reduces the latency to NREM sleep [9, 10]. In mice, localized skin warming from brown adipose tissue thermogenesis could similarly induce NREM sleep [13, 42].

The synaptic pathway for how skin warmth initiates sleep would start with sensory TRPM2 ion channels in the skin that are stimulated by mild (non-painful) external warming [43]. From there, the excitatory pathway would ascend via the lateral parabrachial nucleus [16, 23]. In particular, glutamatergic neurons in the lateral parabrachial nucleus convey signals for temperature place preference [24], and these cells are likely to be involved in our thermal place preference protocol (see Figure 1). From the lateral parabrachial nucleus, the glutamatergic pathway continues to the MnPO-MPO hypothalamus where, in turn, the nitrergic/glutamatergic neurons would be activated (Figure 6). These cells would then excite two groups of neurons: the GABAergic cells recently defined in the lateral preoptic area that elicit hypothermia [25] and the sleep-promoting GABAergic cells in the MPO area identified here in our study. In this way, external warmth, sleep induction, and body cooling would be actively linked (Figure 6).

The glutamate neurons in the MnPO-MPO area are likely to form a key node that links energy expenditure and sleep. Given the small brain volume occupied by the MnPO, we suggest that the glutamate neurons studied by others that regulate body temperature in MnPO are the same nitrergic/glutamatergic cells studied by us that regulate sleep *and* temperature. Our proposed circuit in Figure 6 would explain earlier findings that injecting the GABA_A_ receptor antagonist picrotoxin into the MPO area of rats triggers wakefulness and body warming [44]. Dominant inhibition onto the nitrergic/glutamatergic neurons would produce these effects (Figure 6). It seems highly likely that these nitrergic/glutamatergic cells also express leptin receptors (LepRb), as LepRb-expressing neurons in the MnPO area that cause body cooling when activated are glutamatergic [28, 45]. Depending on context, leptin can often produce inhibitory electrophysiological responses on target neurons [46, 47]. Circulating leptin released from adipose tissue might inhibit the glutamatergic MPO/MnPO cells and would consequently induce both wakefulness and body warming (Figure 6) and therefore increase energy expenditure. Such a mechanism would put these glutamatergic MnPO cells at a nodal point of metabolic regulation.

GABAergic neurons in the MnPO-MPO area are usually thought to be the “prime movers” that induce NREM sleep [3, 18, 37]. For fever, it is proposed that prostaglandin D2 activates cells in the meninges to release adenosine, which in turn activates GABAergic cells in the ventral lateral preoptic nucleus to induce sleep [37]. And while GABAergic neurons in the preoptic area can induce NREM sleep [48], including the GABAergic cells we have identified here (Figure 6), we emphasize that it is actually glutamatergic neurons in the MnPO area that are activated for NREM sleep induction, at least for inducing sleep in response to external warming. On the other hand, if there was a way to selectively pharmacologically stimulate the MPO GABAergic cells that induce NREM sleep without core body cooling (Figure 6), this would be highly desirable in clinical anesthesia, where the pathological consequences of anesthetic-induced hypothermia can be serious [49].

In summary, we have discovered nitrergic/glutamatergic neurons in the MnPO hypothalamus that respond to skin warming and control both NREM sleep induction and body cooling. It has been hypothesized before that cooling the brain is part of the function of NREM sleep [12] and also that a general function of sleep is to conserve energy [50] or, more precisely, to allocate energy to optimize reproduction [51]. Because NREM sleep induction and reduced energy expenditure (no heat production) are inextricably bound together at the circuit level, our work suggests that reduced energy expenditure in the body and/or brain cooling may be an important element of why we sleep.

## STAR★Methods

### Key Resources Table

**Table:** 

REAGENT or RESOURCE	SOURCE	IDENTIFIER
**Antibodies**		

Anti-EGFP rabbit polyclonal antibody	Thermo Fisher Scientific	A6455
Anti-mCherry mouse monoclonal antibody	Clontech	632543/AB_2307319
Mouse monoclonal anti-NOS1	Santa Cruz Biotechnology	sc-5302
Mouse monoclonal anti-NOS1	Sigma	N2280
Rabbit polyclonal anti-parvalbumin	Abcam	ab11427
Rabbit polyclonal anti-calretinin	Abcam	ab702
Rat monoclonal anti-somatostatin	Merck Millipore	MAB354
Guinea Pig anti-vGlut2	Merck Millipore	AB2251-I
Goat polyclonal anti-choline acetyl transferase	Merck Millipore	ab144P
Mouse monoclonal anti-GAD67	Merck Millipore	MAB5406
Rabbit polyclonal anti-c-Fos	Santa Cruz Biotechnology	sc-52
Mouse monoclonal anti-mCherry	Clontech	632543
Rat monoclonal anti-mCherry	Thermo Fisher Scientific	M11217
Alexa Fluor 488 goat anti-rabbit IgG	Molecular Probes	A11034/AB_2576217
Alexa Fluor 594 goat anti-mouse IgG	Molecular Probes	A11005/AB_141372
Alexa Fluor 594 goat anti-rabbit IgG	Molecular Probes	A11037
Alexa Fluor 594 goat anti-rat IgG	Molecular Probes	A11007
Alexa Fluor 488 goat anti-mouse IgG	Molecular Probes	A11001
Alexa Fluor 488 goat anti- Guinea Pig IgG	Thermofisher	A11073
Alexa Fluor 488 donkey anti-goat IgG	Thermofisher	A11055

**Bacterial and Virus Strains**		

AAV-Pcfos-tTA	This paper	
AAV-PTRE-tight-hM3Dq-mCherry	This paper	
AAV-PTRE-tight-flex-hM3Dq-mCherry	This paper	

**Critical Commercial Assays**		

TSA Kit #2, with HRP—Goat Anti-Mouse IgG and Alexa Fluor 488 Tyramide	Thermo Fisher Scientific	T20912

**Chemicals, Peptides, and Recombinant Proteins**		

Isoflurane	Zoetis	50019100
Clozapine *N*-Oxide	Tocris	4936
Doxycycline diet (200 ppm)	Envigo	TD.09265
Doxycycline diet (1000 ppm)	Envigo	TD.09295

**Experimental Models: Cell Lines**		

HEK293 cells	Sigma-Aldrich	**85120602/**CVCL_0045

**Experimental Models: Organisms/Strains**		

Mouse: *Vgat-ires-Cre: Slc32a1*^*tm2(cre)Lowl*^/*J*	[52]	JAX stock 016962
Mouse: *Nos1-ires-Cre*^*tm1(cre)Mgmj*^*/J*	[45]	JAX stock 017526
Mouse: C57BL/6J	Charles River	N/A

**Oligonucleotides**		

Nos1 and Vgat mouse primers	JAX stock	Primers as recommended

**Recombinant DNA**		

Adenovirus helper plasmid *pFΔ6*	Donated by M Klugmann	N/A
AAV helper plasmid *pH21* (AAV1)	Donated by M Klugmann	N/A
AAV helper plasmid pRVI (AAV2)	Donated by M Klugmann	N/A
pAAV-ITR-PcFos-tTA-WPRE-pA-ITR	[32]	Addgene #66794
pAAV-ITR-PTRE-tight-hM3Dq-mCherry-WPRE-pA-ITR	[32]	Addgene #66795
pAAV-ITR-PTRE-tight-flex-hM3Dq-mCherry-WPRE-pA-ITR	This paper	Addgene: Awaiting release

**Software and Algorithms**		

Spike2 v7.18	Cambridge Electronic Design	http://ced.co.uk/products/spkovin
MATLAB (Version R2016b)	MathWorks	https://uk.mathworks.com/
Downloader (Version 1.27)	Evolocus	http://www.evolocus.com
ImageJ	Fiji	https://fiji.sc/
Activity Monitor Version 5 for mice	Medical Associates	http://www.med-associates.com/product-category/activity-software/
Origin Pro 2017	OriginLab	https://www.originlab.com/

**Other**		

1-ml HiTrap Heparin column	Sigma-Aldrich	5-4836
Amicon Ultra-4	Millipore	UFC810024
Angle Two stereotaxic frame	Leica Microsystems	N/A
Hamilton microliter 10-μl syringes	Hamilton	701
Neurologger 2A	[53, 54]	N/A
Temperature logger	Star-Oddi	DST Nano-T
Thermocouples	OMEGA Engineering	SRTC-TT-TI-40-1M
Infrared thermometer	Testo	845
Nestlets - 5cm x 5cm	LBS-biotech	1034011

### Contact for Reagent and Resource Sharing

Further information and requests for resources and reagents should be directed to and will be fulfilled by the Lead Contact, Nicholas P. Franks (n.franks@imperial.ac.uk)

### Experimental Model and Subject Details

#### Mice

Experiments were performed under the Home Office Animal Procedures Act (1986), UK and were approved by local ethics committee. Mice were 10-12 weeks old at the time of the first surgery. The following types of mice were used: *Vgat-ires-Cre: Slc32a1*^*tm2(cre)Lowl*^/*J* (JAX labs stock 016962), kindly donated by Bradford B. Lowell [52]; *Nos1-ires-Cre*^*tm1(cre)Mgmj*^*/J* (JAX labs stock 017526) kindly donated by Martin G Myers [45], and *C57BL/6J* (supplied by Charles River UK). All mice used in the experiments were male and congenic on the *C57BL/6J* background. Mice were maintained on a reversed 12 hr:12 hr light:dark cycle at constant temperature and humidity with *ad libitum* food and water.

### Method Details

#### AAV transgenes

We described previously the construction of the AAV-pan-neuronal activity-tagging system, comprising *pAAV-ITR-P*_*cFos*_*-tTA-WPRE-pA-ITR* (deposited as Addgene plasmid #66794, under Wisden plasmids) and *pAAV-ITR-P*_*TRE-tight*_*-hM3D*_*q*_*-mCherry-WPRE-pA-ITR* (deposited as Addgene plasmid #66795, under Wisden plasmids) transgenes packaged into AAV [32]. For the cell-type-selective version of activity tagging, the *hM3D*_*q*_*-mCherry* reading frame was inverted between heterologous pairs of lox sites (FLEX switch) downstream of the *P*_*TRE-tight*_ promoter (Clontech, Saint-Germain-en-Laye, Paris, France). To make this plasmid, the promoter *P*_*TRE-tight*_ fragment was cut out and isolated from *pAAV-P*_*TRE-tight*_*-hM3D*_*q*_*-mCherry* plasmid, using MluI and SalI restriction enzymes. The plasmid *pAAV-hSyn-flex-hM3D*_*q*_*-mCherry* (gift from Bryan L. Roth, Addgene plasmid #44361) [55] was double digested with MluI and SalI to remove the *hSynapsin* promoter. The *P*_*TRE-tight*_ promoter was ligated into the backbone between MluI and SalI sites to give *pAAV-P*_*TRE-tight*_*-flex-hM3D*_*q*_*-mCherry-WPRE-pA* (deposited at Addgene, awaiting release).

#### Generation of recombinant AAV particles

All AAV transgenes were packaged into AAV capsids (mixed serotype 1 & 2, 1:1 ratio of AAV1 and AAV2 capsid proteins with AAV2 ITRs) as described previously [32].

#### Surgeries and stereotaxic injections of AAV

The mice needed three types of surgery: stereotaxic injections of AAV viruses, ECoG electrode placement (see “EEG and EMG recordings and scoring of vigilance states” below) and temperature logger placement into the abdomen (see “Core Temperature recordings” below). Two of these surgeries, electrode placement and AAV injections, were often performed together. One day before surgery and AAV injection, mice were place on a 200 mg/kg doxycycline (Harlan TD.09265) diet. For surgery, mice were anesthetized with 2% isoflurane. The two viruses required for activity-tagging, *AAV-P*_*cfos*_*-tTA* and *AAV-P*_*TRE-tight*_*-hM3D*_*q*_*-mCherry* (or *AAV-P*_*TRE-tight*_*-flex-hM3D*_*q*_*-mCherry*), were mixed in a 1:1 ratio. Viral infusions, using either a steel injector (10 μl-Hamilton #701) or a silica micro-capillary made in house, were performed with an electronic pump and optimized for the target with up to two injections of 0.15 μL at 0.1 μL min^-1^. The injection coordinates relative to Bregma were AP +0.34 mm, ML 0 mm, DV −4.8 & 5.2. Mice were given three weeks for recovery before the activity-tagging, still keeping the doxycyline diet until the activity-tagging (see below). The week after the first surgery, the temperature loggers were often implanted.

#### EEG and EMG recordings and scoring of vigilance states

EEG, EMG and acceleration were recorded wirelessly using a Neurologger 2A as described previously [53, 54]. Electrodes were placed at Reference - AP +1.5 mm, ML −1.5 mm relative to Bregma, 1st - AP −1.5 mm, ML +1.5 relative to Bregma, 2nd Lambda −1.0 mm, ML 0.0 mm. Four data channels were recorded at a sampling rate of 200 Hz with four times oversampling. The EEG data were downloaded and waveforms visualized using Spike2 software (Cambridge Electronic Design, Cambridge, UK) or MATLAB (MathWorks, Cambridge, UK). The EEG was high-pass filtered (0.5 Hz, −3dB) using a digital filter and the EMG was band-pass filtered between 5-45 Hz (−3dB). Power in the delta (1-4 Hz) and theta (6-9 Hz) bands were calculated, together with the RMS value of the EMG signal (averaged over 5 s), and these were used to define the vigilance states of Wake, NREM and REM with an automatic script [56]. Each vigilance state was then checked and confirmed manually. The RMS (root mean square) amplitude and peak frequency during NREM reduced monotonically with body temperature, with a Q_10_ of 2.0 and 1.9, respectively. This was quantified by measuring the average RMS amplitude as a function of temperature during NREM epochs (Figure S5A), and by measuring the peak frequency of the Fourier transform power spectrum during NREM epochs (Figure S5B). Despite these reductions in amplitude and frequency with body temperature, the vigilance states of Wake, NREM and REM could be unambiguously assigned, as has been reported previously [34].

#### Temperature recordings

Core body temperature was measured using an (abdominally-implanted) temperature logger (DSTnano, Star-Oddi, Herfølge, Denmark) recording on a pre-defined program sampled every two minutes for CNO injections. Skin temperature was measured using a Testo 845 infrared thermometer (head) and a 40-gauge, T-type thermocouple (tail), sampled at 1Hz.

#### Temperature preference test

Mice were habituated to a new cage for at least seven days and allowed to complete a nest using pulped cotten fiber nesting material (Nestlets, Ancare, Bellmore, NY). The mice had *ad libitum* access to food and water. At the end of this habituation period, and two hours before the end of the “Lights off” period, the cage was then placed on temperature-controlled warming plates, adjusted such that one end of the cage was at 32 ± 1°C, while the other end of the cage was at the ambient room temperature (22 ± 1°C). The mouse nest was removed and scrunched into a ball and then returned to the middle of the cage. At the end of this two-hour period with the lights off, the lights came on and the position of the mouse recorded for two hours after the light change. At the end of the experiment the position of the mouse and the two largest pieces of bedding were marked. For each trial, the orientation of the plate and cage was randomized. The position of the mouse was recorded using ANY-maze video-recording software (Stoelting Europe, Dublin, Ireland).

#### Activity-tagging with warm stimulus

Mice were maintained on 200 mg kg^-1^ doxycycline (Harlan TD.09265). For activity-tagging, 48 hr prior to the warm or ambient stimuli, the doxycycline was replaced with a doxycycline-free diet. The activity-tagging stimulus was started at ZT 21, and consisted of 2 hr in a warm-box (held at 32 ± 1°C, 55%–75% relative humidity) containing food and water. For the control stimulus, the box was at ambient temperature. Following the activity-tagging stimuli, the diet was immediately substituted for 1 g kg^-1^ doxycycline (HIGH) (Harlan TD.09295) for 24 hr followed by 200 mg kg^-1^ doxycycline indefinitely. 72 hr after the heat or ambient stimulation, mice were given *i.p.* injections of CNO (5 mg/kg, Tocris, Avonmouth, Bristol, UK) or saline under a randomized protocol and the EEG and core body temperature recorded (see above). The injections took place at approximately ZT 20 (i.e., 4 hours before the “Lights on” period).

CNO has recently been characterized as a low affinity but high efficacy ligand at the *hM3D*_*q*_*-mCherry* receptor, and that CNO’s metabolite clozapine is also a potent ligand at this receptor [35]. Because clozapine at 1 mg/kg reduces locomotor activity [35], it is important to control for the effects of CNO’s administration on sleep. We found that CNO at the dose we used (5 mg/kg) produced no sedation compared with saline injections in background strains of mice congenic for the C57BL/6 background (Figure S4). This is consistent with the findings of Gomez et al. [35] who showed no sedative effects at even higher doses of CNO (10 mg/kg). We also found that the effects of CNO were the same at ambient temperature whether the mice were, or were not, on doxyclycline (see Results), so these data were pooled to give CNO controls.

#### Immunohistochemical detection of activity-tagging and endogenous c-FOS expression

For endogenous c-FOS staining following peripheral heating, mice were killed and perfused 30 mins after stimulus. For detecting activity-tagged hM3D_q_-mCherry expression, mice were killed and perfused 4 days after the activity-tagging stimulus. Mice were given pentobarbital anesthesia (100 mg/kg body weight; *i.p.*), and transcardially perfused with 4% paraformaldehyde in phosphate-buffered saline (PBS), pH 7.4. Brains were removed and 35-μm-thick coronal sections cut using a Leica SM 2010R microtome. Free-floating sections were washed in PBS three times for 10 minutes, permeabilized in PBS plus 0.4% Triton X-100 for 30 min, blocked by incubation in PBS plus 10% normal goat serum (NGS), 0.2% Triton X-100 for 1 h (all at room temperature) and subsequently incubated with either a c-FOS polyclonal antiserum (1/2000, SC-52, SantaCruz), an mCherry monoclonal antibody (either 1/2000, 632543, Clontech, Mountain View, CA or 1/1000, M11217, ThermoFisher Scientific, Waltham, MA), rabbit polyclonal calretinin (1/1000, ab702, Abcam, Cambridge, UK), rabbit polyclonal parvalbumin (1/1000, ab11427 Abcam, Cambridge, UK), rat monoclonal somatostatin (1/200, MAB354, EMD Millipore), goat polyclonanl choline acetyl transferase (1/500, ab144P, EMD Millipore), mouse monoclonal NOS1 (1/1000, N2280 Sigma) and/or mouse monoclonal antibody *Nos1* (1/200, incubated for 48 hours, A-11, sc-5302, Santa Cruz), and/or VGLUT2 antisera (1/100, AB2251-I, EMD Millipore) or a GAD67 (1/1000, MAB5406, EMD Millipore) monoclonal antibody. Primary antisera or monoclonal antibodies were diluted in PBS plus 5% NGS (normal goat serum) overnight or for 48 hours at 4°C. The next day, incubated slices were washed three times (each lasting 10 minutes), in PBS and then incubated for 2 h at room temperature in PBS plus 5% NGS with a 1/1000 dilution of a Alexa Fluor® 488 goat anti-rabbit IgG (H+L) (A11034, Molecular Probes®, Eugene, OR) or Alexa Fluor® 594 goat anti-mouse IgG (H+L) (A11005, Molecular Probes®) or Alexa Fluor® 488 goat anti-guinea pig IgG (H+L) (A11073, Molecular Probes®) or Alexa Fluor® donkey anti-goat IgG (H+L) (c, Molecular Probes®) and subsequently washed there times in PBS for 10 min at room temperature. GAD67 and NOS1 were amplified using a tyramide amplification kit (T20912, Thermo Fisher Scientific, Dartford, UK. The sections were mounted on slides in Vectashield with DAPI (H-1200, Vector Laboratories, UK).

##### Counting the number of cells expressing endogenous c-FOS in MnPO/MPO and neocortex following heat stimulation**:**

For the data shown in Figure 2A and Figure S1 (the numbers of cells expressing endogenous c-FOS following ambient or warm treatments, 4 control mice, 4 treatment mice) MnPO c-FOS-expressing cells were counted on 8 to 10 consecutive (35 μm) sections per mouse between Bregma +0.35 to 0.0 mm; MPO c-FOS-expressing cells were counted on 12 to 14 (35 μm) consecutive sections/mouse between Bregma +0.35 to −0.105 mm; neocortex c-FOS- expressing cells were counted on 16 to 24 (35 μm) consecutive sections per mouse between Bregma +0.735 to −0.105 mm.

##### Counting the number of tagged (hM3D_q_-mCherry receptor-expressing) cells that express *GAD67* or *NOS1* in Pan-MnPO/MPO-Activity-Tag mice:

For *Nos1* these data were taken from 7 sections from 3 mice, and for GAD67 these data were taken from 4 sections from 2 mice (Bregma 0.0 to +0.56).

### Quantification and Statistical Analysis

Origin v8.6 was used for statistical analyses. Data collection and processing were randomized or performed in a counter-balanced manner. Data are represented as the mean ± SEM unless otherwise stated in the figure legends. For the behavioral experiments, two-way ANOVA (vigilance state and treatment factors or genotype), or t tests were performed. p values are shown when they are less than 0.05 (_^∗^_p < 0.05, _^∗∗^_p < 0.01, _^∗∗∗^_p < 0.001, _^∗∗∗∗^_p < 0.0001). When multiple comparisons were made, the Bonferroni-Holm post hoc test was applied. Mice were excluded from the analysis if the histology did not confirm significant AAV transgene expression in the MnPO-MPO preoptic hypothalamus, or if the transgene expression had spread beyond the target region. Investigators were not blinded to treatments.

## Supplemental Information

Document S1. Figures S1–S5

Document S2. Article plus Supplemental Information
