# Supplementary material for: A Neuronal Hub Binding Sleep Initiation and Body Cooling in Response to a Warm External Stimulus
Source: Curr Biol. 2018 Jul 23;28(14):2263–2273.e4. doi: 10.1016/j.cub.2018.05.054 (PMC6078908; doi:10.1016/j.cub.2018.05.054)
Supplement: Document S1. Figures S1–S5 [file mmc1.pdf]

Current Biology, Volume *28*

Supplemental Information

A Neuronal Hub Binding Sleep Initiation and Body Cooling in Response to a Warm External Stimulus

Edward C. Harding, Xiao Yu, Andawei Miao, Nathanael Andrews, Ying Ma, Zhiwen Ye, Leda Lignos, Giulia Miracca, Wei Ba, Raquel Yustos, Alexei L. Vyssotski, William Wisden, and Nicholas P. Franks

Figure S1. Associated with Figure 2. Endogenous c-FOS expression increases in the preoptic (PO) hypothalamus but does not change in the cortex in response to external body warming in *C57BL/6* mice.

(A) The number of c-FOS-expressing cells detected by immunocytochemical staining with a c-FOS antibody in the medial preoptic region (MPO) increases after a warm stimulus. Representative images are shown on the left after both ambient (22 ± ˚C) and warm stimuli (32 ± 1˚C) for two hours.

(B) The number of c-FOS-expressing cells in the MPO region increased in response to the warm stimulus ($p = 0.019$, $df = 6$, $n = 4$). *$p < 0.05$.

(C) The number of c-FOS-expressing cells in the neocortex (Crtx) did not change after a warm stimulus. Representative images are shown on the left after both ambient (22 ± ˚C) and warm stimuli (32 ± 1˚C) for two hours.

(D) The number of c-FOS-expressing cells in the Crtx did not change significantly in response to the warm stimulus ($p = 0.28$, $df = 6$, $n = 4$).

3v, third ventricle; M2, premotor cortex; Cg1, cingulate cortex, subregion 1. The scale bar represents 200 μm. The error bars in B and D represent SEM.

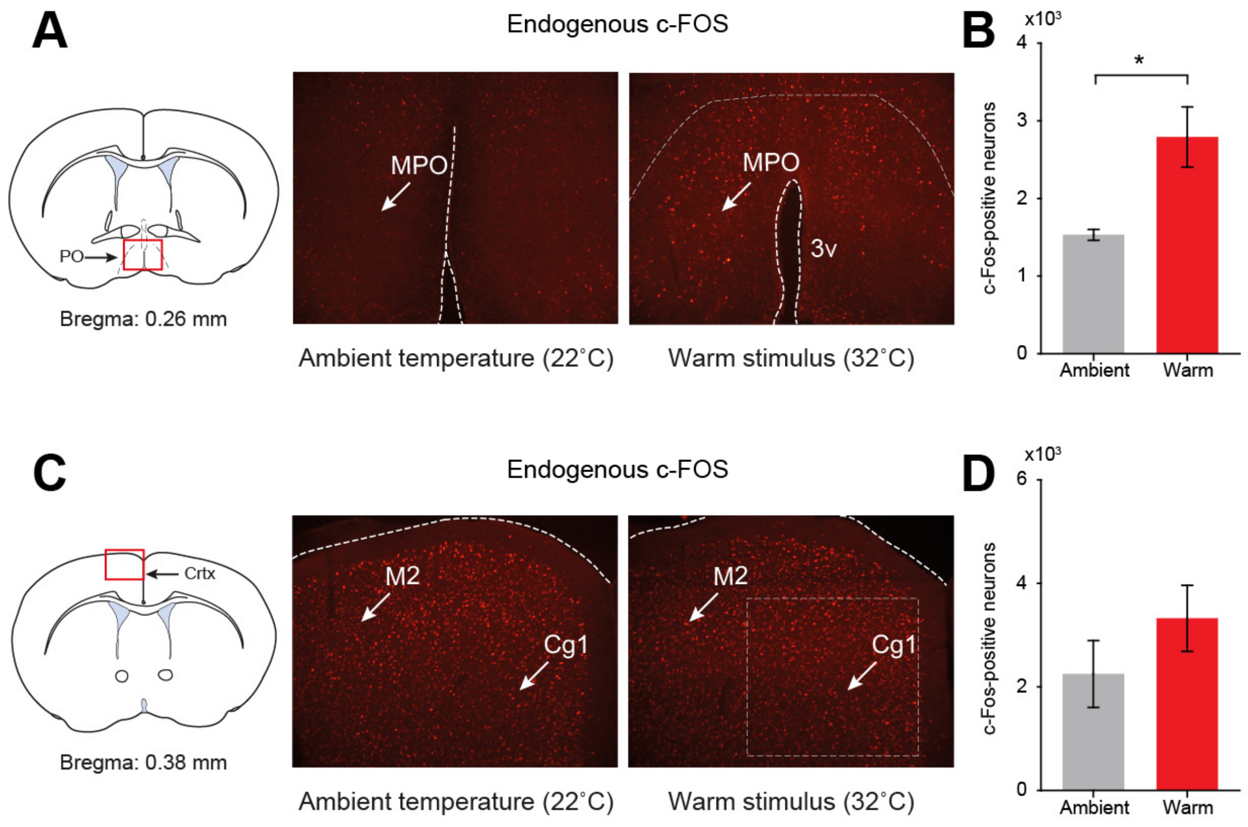
A
Endogenous c-FOS
PO
Bregma: 0.26 mm
MPO
MPO
3v
Ambient temperature (22˚C)
Warm stimulus (32˚C)
B
x10³
c-Fos-positive neurons
4
3
2
1
0
*
Ambient
Warm
C
Endogenous c-FOS
Crtx
Bregma: 0.38 mm
M2
Cg1
M2
Cg1
Ambient temperature (22˚C)
Warm stimulus (32˚C)
D
x10³
c-Fos-positive neurons
6
4
2
0
Ambient
Warm

Figure S1

Figure S2. Associated with Figure 3. Validation of the transgenes used to activity tag warm-sensitive neurons.

(A) HEK293 cells were transformed with P_{cFos}-*tTA* and $P_{TRE\text{-}tight}$-*hM3D*$_q$-*mCherry* transgenes and expression of mCherry observed both with (left-hand image) and without (right-hand image) doxycycline (500 ng/ml) (red indicates hM3D$_q$-mCherry immunocytochemical staining with an mCherry antibody). The insets to the two figures show bright-field images. The scale bars represent 200 μm.

(B) For tet-tagging, these transgenes were packaged in AAV and injected into the MnPO/MPO region of the hypothalamus.

(C) Representative images showing mCherry expression in MnPO and MPO after exposure to ambient temperatures (22 ± 1˚C). The scale bars represent 200 μm.

(D) Representative images showing mCherry expression in MnPO and MPO after exposure to a warm stimulus (32 ± 1˚C).

(E) Higher power images of MnPO neurons stained to show cell nuclei (blue – DAPI staining) and activity-tagged neurons (red). The scale bar represents 20 μm.

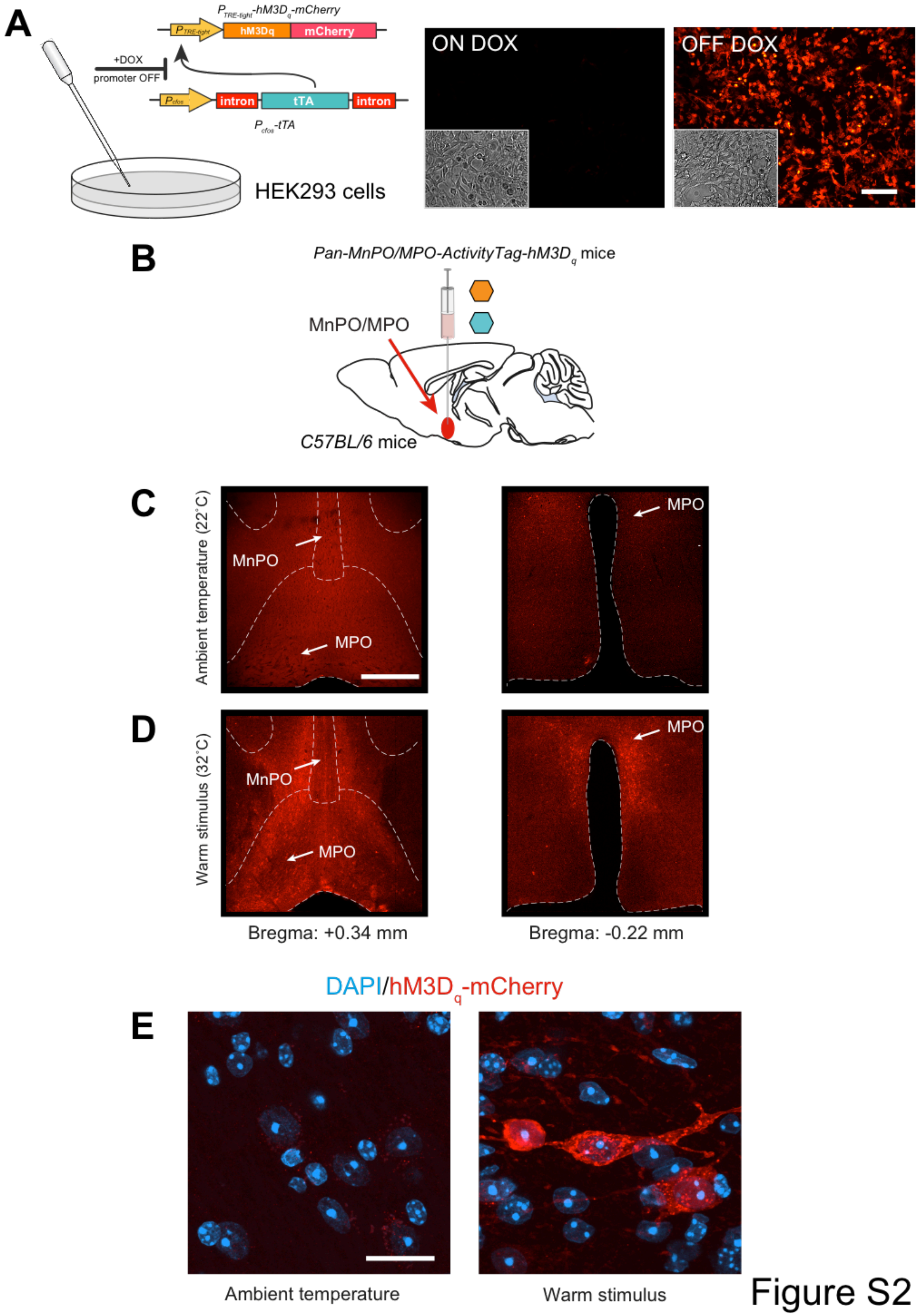
A
P TRE-tight -hM3D q -mCherry
hM3Dq
mCherry
+DOX
promoter OFF
intron
tTA
intron
P cfos -tTA
HEK293 cells
ON DOX
OFF DOX
B
Pan-MnPO/MPO-ActivityTag-hM3D q mice
MnPO/MPO
C57BL/6 mice
C
Ambient temperature (22˚C)
MnPO
MPO
MPO
D
Warm stimulus (32˚C)
MnPO
MPO
MPO
Bregma: +0.34 mm
Bregma: -0.22 mm
DAPI/hM3D q -mCherry
E
Ambient temperature
Warm stimulus

Figure S2

Figure S3. Associated with Figure 3. Anatomical distribution of warm-stimulus-tagged neurons in the MnPO/MPO hypothalamus.

(A) AAV-$P_{TRE\text{-}tight}$-*hM3D*$_q$-*mCherry* and AAV-P_{cfos}-*tTA* were injected in the MnPO/MPO hypothalamus, and neurons were tet-tagged following a warm stimulus in *C57BL/6* (*n* = 11) mice.

(B) Expression of mCherry was mapped using an mCherry antibody rostral-caudal across the injection region. These images were then aligned and overlapped and a common region of expression was delineated.

(C) The common region of expression of warm-sensitive tet-tagged neurons across the injection region.

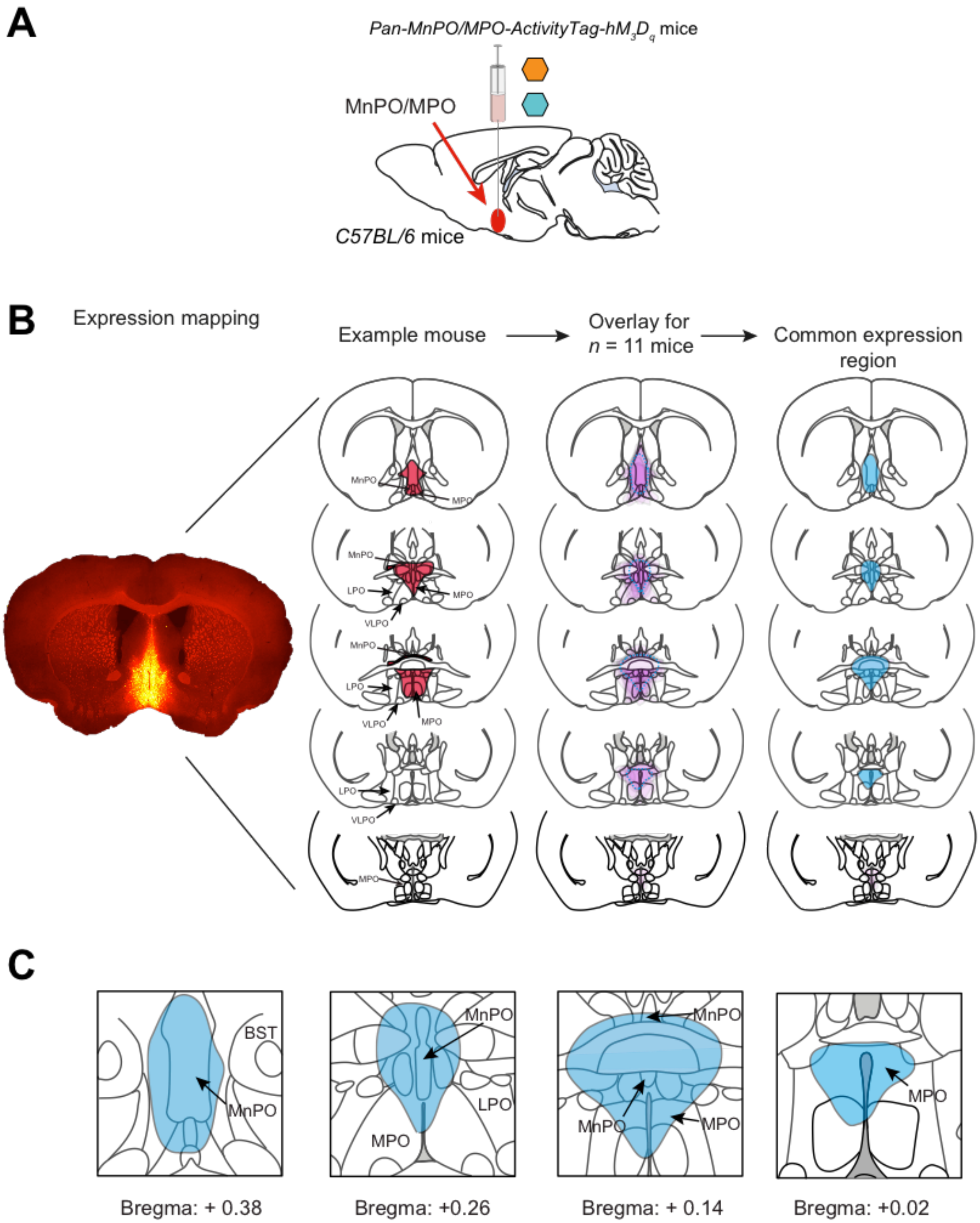
A
Pan-MnPO/MPO-ActivityTag-hM3Dq mice
MnPO/MPO
C57BL/6 mice
B
Expression mapping
Example mouse
Overlay for n = 11 mice
Common expression region
MnPO
MPO
LPO
VLPO
C
BST
MnPO
LPO
MPO
Bregma: + 0.38
Bregma: +0.26
Bregma: + 0.14
Bregma: +0.02

Figure S3

Figure S4. Associated with Figure 3. Control CNO experiments.

(A) The lowest body temperatures of mice after a CNO injection (5 mg/kg) following exposure to ambient temperature was not significantly difference whether the mouse was on (green symbols; *n* = 6) or off (grey symbols; *n* = 7) doxycycline (200 mg/kg) ($p = 0.25$, df = 11). These data were pooled to establish the control CNO data. This temperature was significantly different ($p = 4.35 \times 10^{-7}$; df = 25) to the lowest body temperatures of mice after a CNO injection following exposure to a warm stimulus (red symbols; *n* = 14).

(B) The percentage of NREM (1-hr average, 40 minutes after CNO injection) of mice after a CNO injection following exposure to ambient temperature was not significantly difference whether the mouse was on (green symbols; *n* = 10) or off (grey symbols; *n* = 12) doxycycline (200 mg/kg) ($p = 0.67$, df = 20). These data were pooled to establish the control CNO data. These data were also not significantly different to the percentage of NREM after a CNO injection following exposure to ambient temperature, but for mice where no tet-tagging viruses had been injected into the MnPO/MPO (brown symbols; *n* = 8) ($p = 0.10$, df = 28).

(C) CNO injections (5 mg/kg) did not change the percentage of NREM (1-hr average, 40 minutes after CNO injection. Grey symbols; *n* = 14) above percentage of NREM observed after saline injection (yellow symbols; *n* = 17).

The error bars represent SEM. ***$p < 0.001$, ****$p < 0.0001$.

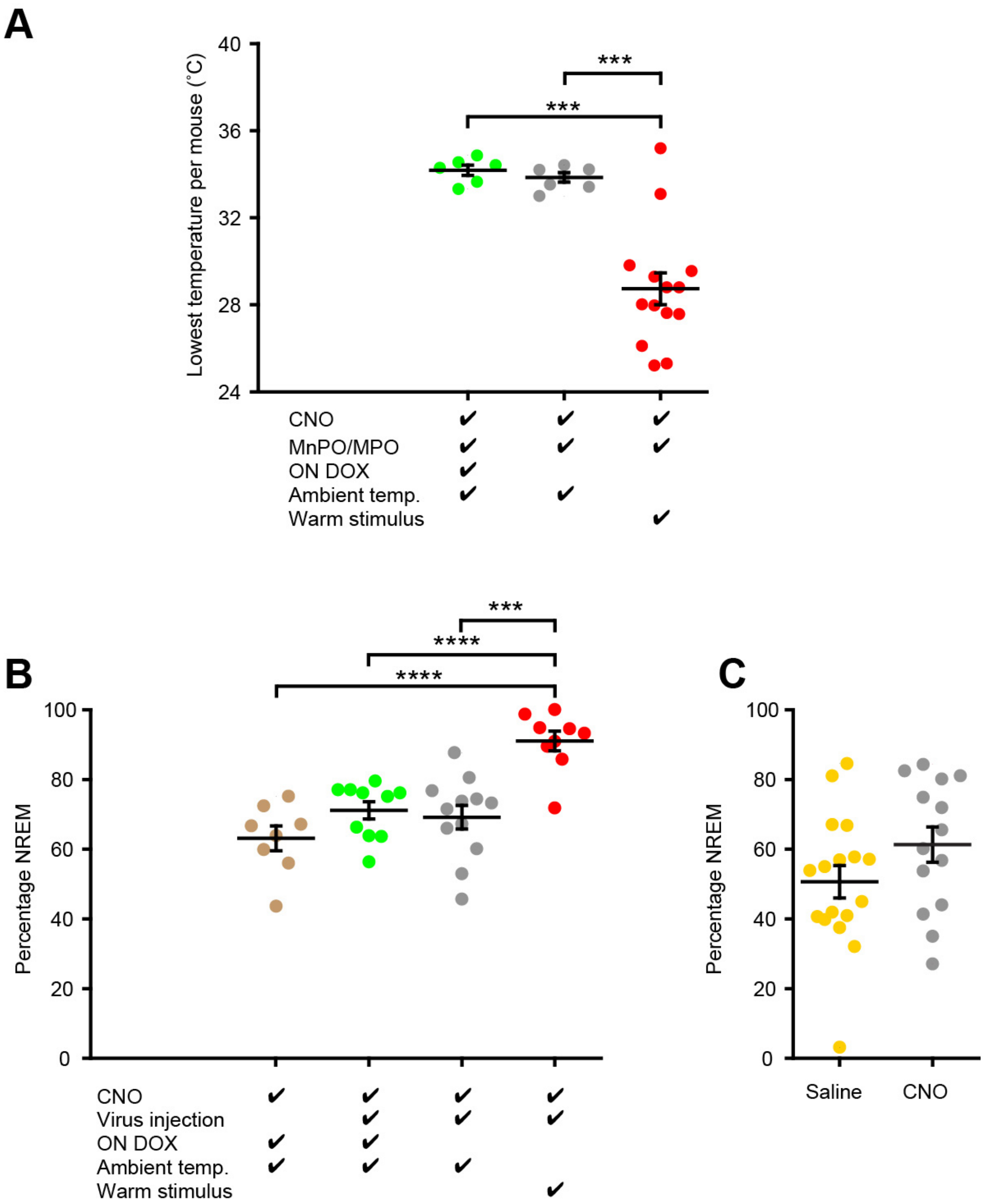
A
Lowest temperature per mouse (˚C)
40
36
32
28
24
***
***
CNO
MnPO/MPO
ON DOX
Ambient temp.
Warm stimulus
B
Percentage NREM
100
80
60
40
20
0
***
****
****
CNO
Virus injection
ON DOX
Ambient temp.
Warm stimulus
C
Percentage NREM
100
80
60
40
20
0
Saline
CNO

Figure S4

Figure S5. Associated with Figure 3. The root mean square amplitude of the EEG signal decreased, and the peak of the delta frequency band in the EEG also decreased, as the core body temperature decreased during the hypothermia induced by CNO in warm-stimulated, activity-tagged, *Pan-MnPO/MPO-ActivityTag-hM3D*$_q$ mice (n = 7). (**A**) EEG RMS amplitude during NREM plotted as a function of core body temperature. The data were normalized such that the average RMS value for the EEG at 30 ± 0.5˚C for each animal was unity. The Q_{10} (increase over 10˚C) was 2.0. (**B**) Peak delta (1 – 4 Hz) frequency during NREM plotted as a function of core body temperature. The Q_{10} (increase over 10˚C) was 1.9. The red lines represent the 95% confidence intervals.

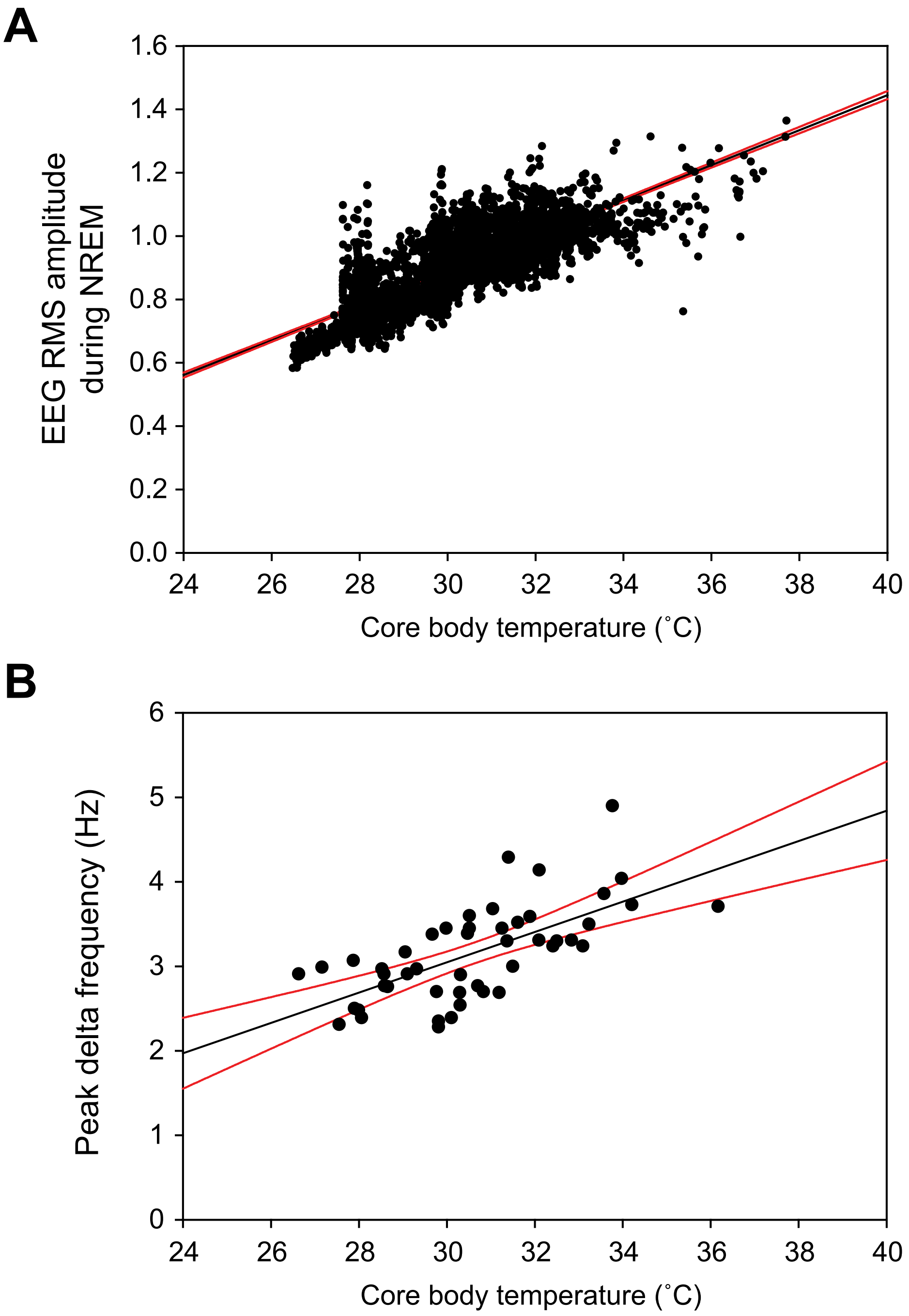
A
1.6
1.4
1.2
1.0
0.8
0.6
0.4
0.2
0.0
EEG RMS amplitude during NREM
24
26
28
30
32
34
36
38
40
Core body temperature (˚C)
B
6
5
4
3
2
1
0
Peak delta frequency (Hz)
24
26
28
30
32
34
36
38
40
Core body temperature (˚C)

Figure S5
